# Integration of SARS-CoV-2 testing and genomic sequencing into influenza sentinel surveillance in Uganda, January to December 2022

**DOI:** 10.1128/spectrum.01328-23

**Published:** 2023-10-09

**Authors:** John T. Kayiwa, Charity Nassuna, Sophia Mulei, Gladys Kiggundu, Joweria Nakaseegu, Maria Nabbuto, Esther Amwine, Bridget Nakamoga, Sarah Nankinga, Phiona Atuhaire, Pheobe Nabiryo, Pixy Alunzi, Tony Mbaziira, Paul Isabirye, Noel Ayuro, Nicholas Owor, Jocelyn Kiconco, Barnabas Bakamutumaho, Earl Austin Middlebrook, Pontiano Kaleebu, Julius J. Lutwama, Andrew William Bartlow

**Affiliations:** 1 Department of Arbovirology, Emerging and Re-emerging Viral Diseases, Uganda Virus Research Institute, Entebbe, Uganda; 2 Genomics and Bioanalytics, Los Alamos National Laboratory, Los Alamos, New Mexico, USA; 3 Medical Research Council/Uganda Virus Research Institute & London School of Hygiene & Tropical Medicine, Uganda Research Unit, Entebbe, Uganda; Quest Diagnostics, San Juan Capistrano, California, USA

**Keywords:** ILI/SARI surveillance, genomics, NGS, bioinformatics, diagnostics, sentinel surveillance

## Abstract

The Uganda Virus Research Institute, National Influenza Center laboratory integrated SARS-CoV-2 polymerase chain reaction testing and genomic sequencing into the influenza surveillance program that was established in 2007. A total of 7,698 nasopharyngeal/oropharyngeal (NP/OP) swab samples were collected and analyzed from ILI/SARI sentinel sites across the country from January to December 2022. All samples were tested for influenza and SARS-CoV-2. Of these, 252 (3.3%), 162 (2.1%), and 589 (7.7%) were positive for influenza A, influenza B, and SARS-CoV-2, respectively. Out of 414 influenza-positive samples, 122 (29.5%) were AH1pdm09, 130 (31.4%) were AH3, and 162 (39.1%) were B-Victoria. All SARS-CoV-2 sequenced samples were of the Omicron variant, with subvariants of concern known to evade the immune system being detected, such as BQ.1 and XBB.2. Other SARS-CoV-2 positive samples collected from other health centers in the community outside the surveillance sites were included into SARS-CoV-2 genomic sequencing with similar patterns with respect to variants. In all, the ILI/SARI surveillance system has shown to be an efficient, cost-effective, and sustainable program, providing a ready platform to monitor the circulation of SARS-CoV-2 in communities at the national level while remaining vigilant for the persistent threat of influenza. The integration of SARS-CoV-2 detection and genomic surveillance into the influenza surveillance program will strengthen the laboratory response capacity, as well as facilitate the timely release of SARS-CoV-2 genomic information to be used to complement the multiple response strategies for COVID-19 pandemic mitigation.

**IMPORTANCE:**

Respiratory pathogens cause high rates of morbidity and mortality globally and have high pandemic potential. During the SARS-CoV-2 pandemic, influenza surveillance was significantly interrupted because of resources being diverted to SARS-CoV-2 testing and sequencing. Based on recommendations from the World Health Organization, the Uganda Virus Research Institute, National Influenza Center laboratory integrated SARS-CoV-2 testing and genomic sequencing into the influenza surveillance program. We describe the results of influenza and SARS-CoV-2 testing of samples collected from 16 sentinel surveillance sites located throughout Uganda as well as SARS-CoV-2 testing and sequencing in other health centers. The surveillance system showed that both SARS-CoV-2 and influenza can be monitored in communities at the national level. The integration of SARS-CoV-2 detection and genomic surveillance into the influenza surveillance program will help facilitate the timely release of SARS-CoV-2 information for COVID-19 pandemic mitigation and provide important information regarding the persistent threat of influenza.

## INTRODUCTION

Globally, infections caused by respiratory pathogens account for high morbidity and mortality (1 – 4). Prevention and control are a challenge due to their high transmissibility and ability to quickly evolve, making them likely candidates for epidemics and pandemics. Such is exemplified by the occurrence of two respiratory virus pandemics in the last two decades: influenza A/H1N1pdm09 in 2009 and SARS-CoV-2 in 2020. Both viruses spread to almost all parts of the world in a very short time, caused primarily by international travel (5). Influenza A/H1N1pdm09 cases and deaths continued to occur every year after its emergence, with varying seasonal peaks (6) and other epidemiological factors.

Starting in January 2020 with the emergence and spread of SARS-CoV-2, influenza surveillance systems across the world were significantly disrupted due to the diversion of resources and supplies to SARS-CoV-2 testing. This also led to the reduction in the number of influenza cases reported globally (7, 8). The reasons in the reduction of influenza virus transmission may have been due to COVID-19-related non-pharmaceutical interventions (8) to curb the SARS-CoV-2 pandemic, such as the implementation of curfews, restriction of public and social gatherings, mask-wearing in public places, and emphasis of online meetings. The lack of reliable and affordable multiplex diagnostic kits for the detection of respiratory viruses may also have posed a challenge for syndromic testing (9).

The emergence of novel SARS-CoV-2 strains raised the need to monitor the genomic variations by specialized laboratories with sequencing capacity, as any gap could lead to serious public health consequences. Another major and immediate public health concern was the lack of reporting pertaining to other respiratory viruses since co-circulating respiratory viruses other than SARS-CoV-2 remained largely undocumented for nearly the entire years of 2020 and 2021. Therefore, reverse transcription polymerase chain reaction (RT-PCR)-based SARS-CoV-2 detection methods needed to be complemented with two additional monitoring measures. The first is sequencing the SARS-CoV-2 genome to identify novel variants that can provide actionable information to prevent or mitigate emerging viral threats. The second is to test for co-circulating respiratory viruses that might be independent factors contributing to disease burden.

Later in the pandemic, the World Health Organization (WHO) produced interim guidance for countries to maintain or restart influenza surveillance as well as to monitor SARS-CoV-2 (10). As such, the implementation of genomic sequencing of SARS-CoV-2 from samples collected from representative sentinel sites was expedited to monitor the trends and prevalence of existing and emerging (co-)circulating genetic variants. This also helped to improve the geographical and demographic representativeness and the timeliness of SARS-CoV-2 genetic sequence data to inform public health interventions.

Case definitions for COVID-19 were reviewed after growing knowledge of the most common and predictive clinical features of COVID-19 were found to be fever (83%) and cough (60%), followed by loss of taste/smell (41%), fatigue (31%), and loss of appetite (30%) (11). These symptoms were included in the current influenza-like illness (ILI) and severe acute respiratory illness (SARI) case definitions. WHO then advocated for influenza sentinel surveillance systems to integrate SARS-CoV-2 testing of specimens collected from influenza surveillance sources, with a recommendation to test both pathogens simultaneously, preferably by multiplex PCR assays (10).

In Uganda, the National Influenza Center (NIC) laboratory at the Uganda Virus Research Institute (UVRI), a WHO-designated NIC, was established in 2007 with the aim of providing information on the seasonality and epidemiology of the influenza virus, giving notifications of outbreaks and novel strains, and providing recommendations on strain selection for the annual formulation of the seasonal influenza vaccine (11). The influenza surveillance program uses all or a selection of cases meeting the case definitions of ILI, which is an acute respiratory infection with a measured fever of ≥38°C and a cough with onset within the last 10 days. The case definition of SARI is an acute respiratory infection with a subjective or measured temperature of ≥38°C and a cough with onset in the last 10 days requiring hospitalization (11).

**Fig 1:**
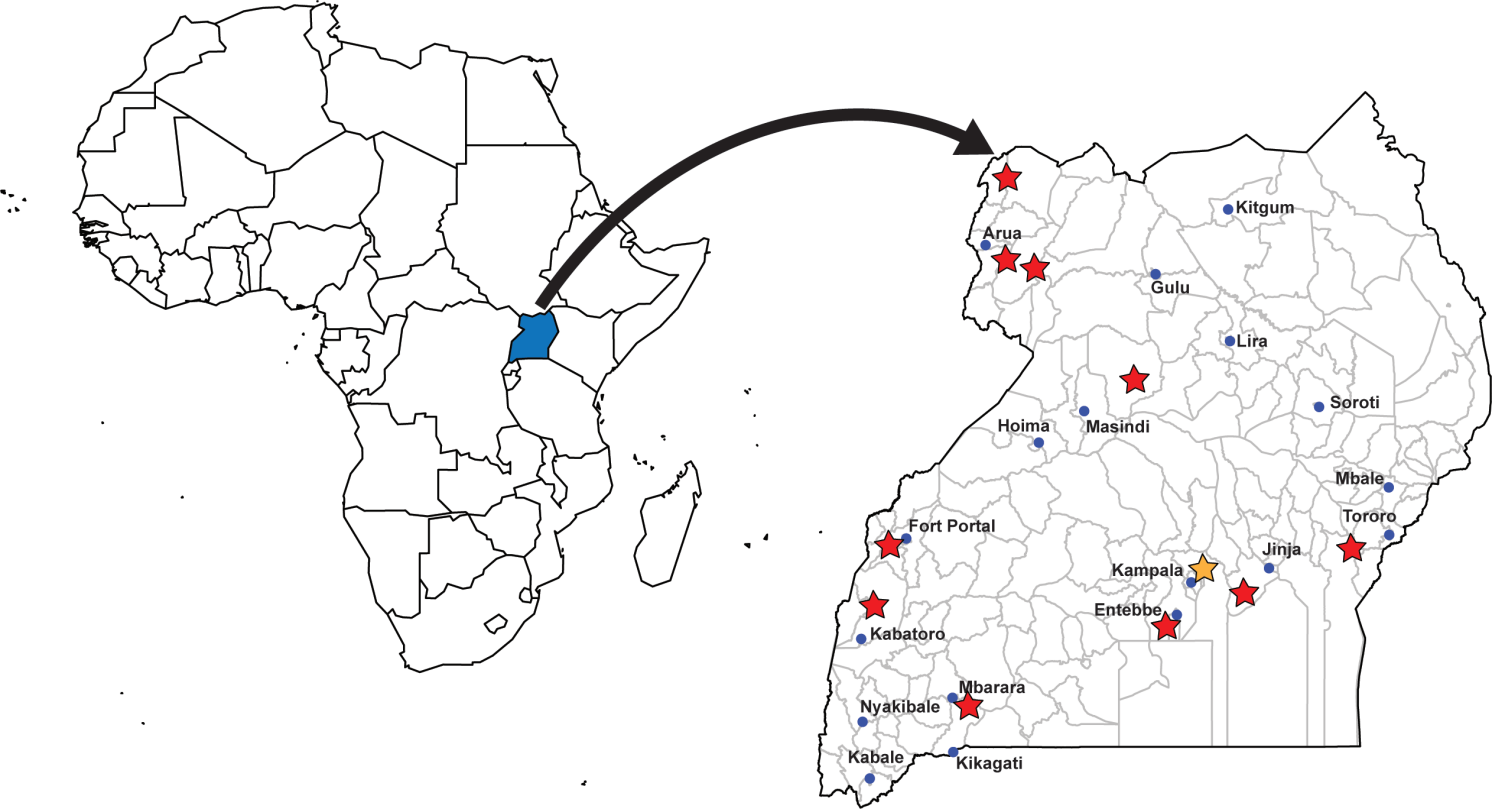
Map of Uganda showing the location of the ILI/SARI sentinel sites. The following sites are designated by a red star: Entebbe Regional Referral Hospital, Mukono General Hospital, Fort Portal Hospital, Mbarara Hospital, Arua Regional Referral Hospital, Bibia Health Center III—Arua, Karambi Health Center III—Kasese, Kiryandongo Hospital, Koboko Hospital, Logiri Health Center III, Tororo General Hospital. Kawaala Health Center IV, Kiswa Health Center III, Kitebi Health Center III, Nsambya Hospital, and Kibuli Hospital are all located in the Kampala district and are designated by the orange star.

The influenza surveillance program comprises a network of 16 sentinel sites geographically spread across the country (Fig. 1). The selected sentinel sites include general hospitals and health centers that provide treatment to all age groups of patients and are in areas with high population density and high trade, where influenza transmission would likely be more rapid during a pandemic. The hospitals offer both outpatient and inpatient care, whereas the health centers offer only outpatient care. The surveillance is conducted in 11 districts representing 4 geographically distinct regions across Uganda (Fig. 1). The UVRI-NIC laboratory therefore conducts and plays an important role in the COVID-19 pandemic response by providing a ready platform to monitor the circulation of SARS-CoV-2 in communities at the national level. The population of the districts in Uganda was reported in 2012 when the initial sentinel surveillance system was established (see Fig. 1 in reference 12). The sites in this system represent major population centers and areas of high trade (12). The ILI/SARI surveillance program is also an efficient, cost-effective, and sustainable platform in response to the COVID-19 pandemic while remaining vigilant for a persistent threat of influenza.

Our work describes the successful integration of SARS-CoV-2 detection and genomic sequencing into the ILI/SARI surveillance system of the WHO UVRI-NIC laboratory for the period of January to December 2022 (Epi week 50). Our main goals were to identify and track the number and proportion of influenza and SARS-CoV-2 infections at the surveillance sites and in other community health centers throughout Uganda, as well as determine SARS-CoV-2 variants through whole-genome sequencing by continuously sampling patients. This integration strengthens the laboratory response capacity, as well as facilitates the timely release of SARS-CoV-2 genomic information to be used to complement the multiple response strategies for COVID-19 pandemic mitigation.

## MATERIALS AND METHODS

### Sample collection and processing

The study used nasopharyngeal (NP) and/or oropharyngeal (OP) swabs collected between January and December 2022 (Epi week 50) from patients visiting ILI/SARI sites and other community health centers outside the sentinel site surveillance program. The influenza surveillance program is a network of 16 sentinel sites located throughout the country (Fig. 1). Samples from other health centers in Uganda represented community-level cases throughout the country. In total, 7,698 samples were collected from ILI/SARI sentinel surveillance sites and 23,167 samples were collected from other health centers throughout Uganda (community-level cases). All samples were tested for SARS-CoV-2, but only the 7,698 samples collected from surveillance sites were also tested for influenza.

RNA was extracted from 140 μL of NP/OP swab specimens in a viral transport medium (VTM) using the QIAamp Mini Viral RNA Extraction Kit from Qiagen (Haldeman, Germany) according to the manufacturer’s instructions. The RNA was tested on the ABI 7500 Real-Time PCR platform for influenza and SARS-CoV-2 using the CDC influenza SARS-CoV-2 (Flu SC2) multiplex Real-Time Reverse Transcription Polymerase Chain Reaction (rRT-PCR) (CDC Influenza Virus Real-Time RT-PCR Influenza A/B Typing Panel (VER 2) (RUO) (Catalog No. FluRUO-14) as per CDC protocol (13). All samples were tested for human RNAse P (RP) gene as a housekeeping gene. The RP gene serves as an internal control for the Flu SC2 Multiplex assay that is compared to other targets for the interpretation of an individual specimen. Briefly, RP should be positive for clinical specimens in the absence of a signal for one of the viral targets. A negative RP in the presence of a positive result for one of the viral targets, the viral target should be considered valid. However, if all viral targets and RP are negative, the test should be considered invalid (13). A second CDC multiplex rRT-PCR was performed for all influenza A positives, to subtype for influenza AH1pm09 and AH3 using the CDC Influenza Virus Real-Time RT-PCR Influenza A (H3/H1pdm09) Subtyping Panel (VER 3) (RUO) (Catalog No. FluRUO-15), whereas influenza B positives were genotyped for influenza B Victoria and Yamagata using the CDC Influenza B Lineage Genotyping Panel (RUO) (Catalog No. FluRUO-11).

### Whole-genome sequencing of SARS-CoV-2

SARS-CoV-2 PCR-positive samples from the surveillance sites with high viral load (CT < 30) were selected for whole-genome sequencing. Additional SARS-CoV-2-positive samples collected from other health centers in Uganda were also selected for whole-genome sequencing. Briefly, sequence libraries were generated from SARS-CoV-2-positive samples using the Swift Normalase Amplicon Panel (SNAP) SARS-CoV-2 kit (14) according to the manufacturer’s protocol and sequenced on the Illumina MiSeq platform (Illumina, San Diego, CA), using the MiSeq Reagents Kit v3 (600 cycles).

### Bioinformatics analysis

All SARS-CoV-2 sequence analysis was conducted using the EDGE COVID-19 (EC-19) pipeline (https://edge-covid19.edgebioinformatics.org) (15). Within EC-19, sequence reads were trimmed for quality with FaQCS (16) using default parameters (quality > 20; min read length > 50; max ambiguous bases < 10). The remaining reads were aligned to human reference (GRCh38) with minimap2 v2.17 (17) and filtered out if they share >95% identity. Swift primers were removed using EC-19’s Align Trim. The remaining reads were aligned to the WUHAN-HU1 SARS-CoV-2 reference genome (NCBI: NC_045512.2) with BWA-MEM v0.7.12 (18) and SNPs/INDELS were called with the EC-19 pipeline (15). Variants at greater than 50% frequency and coverage greater than 5× were used to create consensus genomes for each sample. Resulting SARS-CoV-2 consensus genomes were uploaded to GISAID with accession numbers listed in Table 1. Only those genomes accepted to GISAID are referenced here for which we describe the variants. We report Pangolin lineages from GISAID as of 9 August 2023.

**TABLE 1:** Accession numbers of the SARS-CoV-2 genomes deposited in GISAID (www.gisaid.org)^*a*^

Surveillance sites	Community health centers	
EPI_ISL_16993632	EPI_ISL_18079188	EPI_ISL_16549158
EPI_ISL_16993631	EPI_ISL_18080392	EPI_ISL_18079203
EPI_ISL_16982256	EPI_ISL_18079214	EPI_ISL_18079204
EPI_ISL_16982255	EPI_ISL_18079216	EPI_ISL_18079219
EPI_ISL_17292846	EPI_ISL_18079215	EPI_ISL_18079220
EPI_ISL_17292847	EPI_ISL_17293055	EPI_ISL_18079226
EPI_ISL_17292848	EPI_ISL_15485354	EPI_ISL_18079205
EPI_ISL_17292849	EPI_ISL_15485358	EPI_ISL_18079191
EPI_ISL_17292850	EPI_ISL_15485229	EPI_ISL_18079227
EPI_ISL_17292851	EPI_ISL_15484014	EPI_ISL_18079229
EPI_ISL_17292852	EPI_ISL_15484013	EPI_ISL_18079206
EPI_ISL_17292853	EPI_ISL_15482505	EPI_ISL_18079230
EPI_ISL_17292854	EPI_ISL_18080302	EPI_ISL_18079218
EPI_ISL_17292855	EPI_ISL_15481997	EPI_ISL_18079225
EPI_ISL_17292856	EPI_ISL_18080301	EPI_ISL_18079207
EPI_ISL_18079198	EPI_ISL_15473525	EPI_ISL_18079208
EPI_ISL_17292857	EPI_ISL_18080300	EPI_ISL_18079197
EPI_ISL_18079199	EPI_ISL_15481380	EPI_ISL_18079235
EPI_ISL_17292858	EPI_ISL_15473941	EPI_ISL_18079221
EPI_ISL_17292859	EPI_ISL_18080299	EPI_ISL_18079222
EPI_ISL_17292860	EPI_ISL_15476313	EPI_ISL_18079194
EPI_ISL_18079201	EPI_ISL_15477275	EPI_ISL_18079192
EPI_ISL_18079202	EPI_ISL_15480975	EPI_ISL_18079190
EPI_ISL_18079200	EPI_ISL_15481250	EPI_ISL_18079232
EPI_ISL_17292861	EPI_ISL_18080303	EPI_ISL_18079233
EPI_ISL_17292862	EPI_ISL_15484009	EPI_ISL_18079196
EPI_ISL_16549309	EPI_ISL_15483999	EPI_ISL_18079193
EPI_ISL_17292863	EPI_ISL_15483968	EPI_ISL_18079195
EPI_ISL_17292864	EPI_ISL_15483472	EPI_ISL_18079223
EPI_ISL_17292865	EPI_ISL_15481366	EPI_ISL_18079209
EPI_ISL_17292866	EPI_ISL_15481365	EPI_ISL_18079231
EPI_ISL_17292867	EPI_ISL_15481364	EPI_ISL_18079234
EPI_ISL_17292868	EPI_ISL_15481338	EPI_ISL_18079217
EPI_ISL_17292869	EPI_ISL_16982261	EPI_ISL_18079224
EPI_ISL_17292870	EPI_ISL_16982260	EPI_ISL_18079228
EPI_ISL_17293039	EPI_ISL_16982259	EPI_ISL_17292984
EPI_ISL_17292871	EPI_ISL_16993633	EPI_ISL_17292985
EPI_ISL_17292872	EPI_ISL_16982258	EPI_ISL_17292986
EPI_ISL_17292873	EPI_ISL_16982257	EPI_ISL_17292987
EPI_ISL_16548952	EPI_ISL_16680432	EPI_ISL_17292988
EPI_ISL_16547635	EPI_ISL_16680431	EPI_ISL_17292989
EPI_ISL_18079189	EPI_ISL_16678969	EPI_ISL_17292990
EPI_ISL_17292874	EPI_ISL_16982268	EPI_ISL_17292991
EPI_ISL_17293041	EPI_ISL_16982254	EPI_ISL_18075880
EPI_ISL_17293042	EPI_ISL_16982253	EPI_ISL_18079212
EPI_ISL_17293043	EPI_ISL_16982252	EPI_ISL_18079213
EPI_ISL_17293045	EPI_ISL_16982251	EPI_ISL_18079211
EPI_ISL_16547492	EPI_ISL_18078998	EPI_ISL_18079187
EPI_ISL_16547177		EPI_ISL_18079210

^*a*^ They are split up by those obtained from surveillance sites and other community health centers throughout Uganda.

## RESULTS

**Fig 2:**
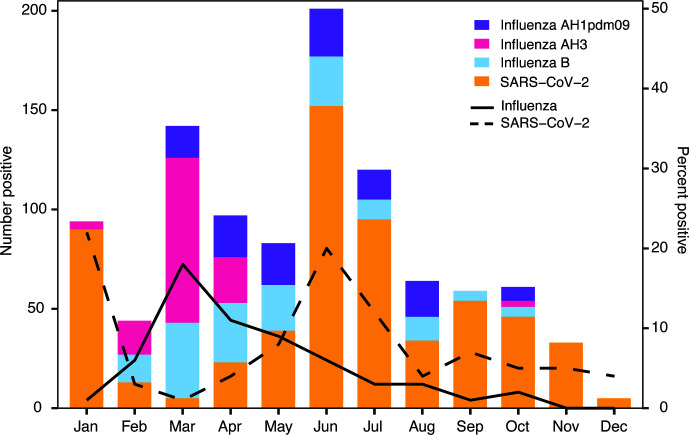
The number of positive cases of SARS-CoV-2 (orange) and influenza (broken up into AH1, AH3, and Influenza B) at the surveillance sites throughout Uganda from January 2022 to December 2022 (Epi week 50). The lines denote the percent positive cases for all influenza cases (solid line) and SARS-CoV-2 (dashed line).

During the study period, a total of 7,698 ILI/SARI sentinel site surveillance samples were collected between January and December 2022 (Epi week 50) and tested by the UVRI-NIC laboratory. Of these, 252 (3.3%), 162 (2.1%), and 589 (7.7%) were positive for influenza A, influenza B, and SARS-CoV-2, respectively (Fig. 2). Of 252 influenza A positives, 130 (51.6%) were AH3 and 122 (48.4%) were AH1pdm09 (Fig. 2). All influenza B positives were of the Victoria lineage.

The monthly influenza and SARS-CoV-2-positive cases for 2022 as captured by the ILI/SARI surveillance program are shown in Fig. 2. The positivity of SARS-CoV-2 among ILI/SARI first peaked in January at 22% decreasing in February and March before peaking again in June at 20% and staying at less than 10% between August and December 2022. By contrast, influenza activity remained low, only peaking in March at 17% and decreasing to less than 10% from May to December 2022. Early in the year, influenza subtype AH3 was the predominate A subtype until May, at which point AH1pdm09 was predominate the rest of the year. AH3 did not appear again until October. Influenza B Victoria was found from February until October.

A total of 22 cases of co-infections from ILI/SARI surveillance sites were detected during the study period: 2 cases with SARS-CoV-2/AH3; 9 cases with SARS-CoV-2/B-Victoria; and 11 cases with SARS-CoV-2/AH1pdm09. The outcome of the 22 SARS-CoV-2/influenza co-infected patients is not known, as the cases were not followed up.

**Fig 3:**
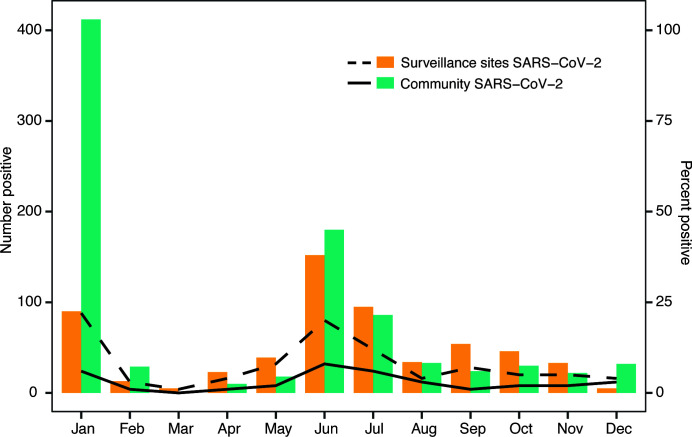
The number of positive cases of SARS-CoV-2 at both the ILI/SARI surveillance sites (orange) and in the community (green) from January 2022 to December 2022 (Epi week 50). The lines denote the percent positive cases of SARS-CoV-2 at surveillance sites (dashed line) and in the community (solid line).

We also tested a total of 23,167 samples collected from other community health centers outside the sentinel sites and found an overall positivity of 3.8% for SARS-CoV-2. These samples were only tested for SARS-CoV-2. A similar trend in positivity by month was observed when ILI/SARI sentinel site data were compared to SARS-CoV-2 testing in samples collected from health centers outside the sentinel site surveillance program at UVRI (Fig. 3). Both sets of samples show peaks in January and again in June 2022.

**Fig 4:**
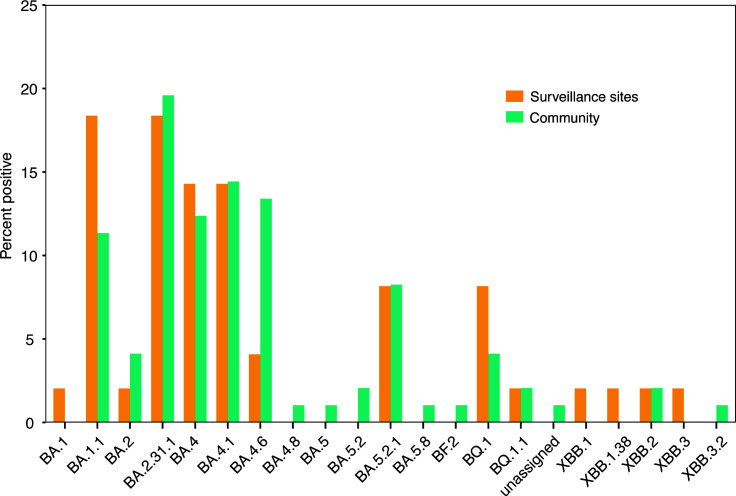
The percent of Omicron lineages and sublineages detected at surveillance sites (orange) and in the community (green) from samples collected from January 2022 to December 2022 (Epi week 50).

Samples were chosen for whole-genome sequencing based on the PCR CT values of samples collected during the reporting period. In total, 49 samples from sentinel surveillance sites and 97 samples from other health centers (community) were successfully assembled into SARS-CoV-2 consensus genomes and publicly shared through GISAID (https://www.gisaid.org), an initiative founded on sharing influenza virus sequencing data (Table 1). Samples failing consensus did so because of greater than 50% ambiguous bases. All 146 genomes were classified as Omicron (Fig. 4). The Omicron subvariant BA.2.31.1 ranked as the predominant SARS-CoV-2 strain during the reporting period for samples collected from other health centers (19/97) (Fig. 4). For surveillance sites, BA.1.1 (9/49) and BA.2.31.1 (9/49) were the predominate strains throughout the year. Generally, there were similar percentages between surveillance sites and other community health centers for each of the variants identified during our sampling period (Fig. 4).

## DISCUSSION

Our main objective was implementing a comprehensive surveillance program for influenza and SARS-CoV-2 following the WHO recommendations to identify and track the proportion and subtypes of influenza and variants of SARS-CoV-2 infections in the community. During the study period, we identified more influenza A viruses compared to influenza B. Our surveillance also demonstrated a possible inverse relationship between influenza and SARS-CoV-2 activity (Fig. 2). However, this observation may be biased as we were not able to account for non-pharmaceutical interventions that were implemented during COVID-19 upsurge, leading to a reduction in influenza transmission. All influenza B viruses belonged to the Victoria lineage, similar to results in Uganda in 2009–2010 (19).

During its inception, our program detected more SARS-CoV-2 cases than influenza in January 2022. This could be attributed to the time of integration of SARS-CoV-2 testing and genomic sequencing into ILI/SARI surveillance in January 2022, during the peak of the third wave of COVID-19 in Uganda. SARS-CoV-2 cases peaked during June 2022 with a similar percent positivity in January. We observed an increase in influenza activity during the early months of 2022, which was different from the seasonality of influenza previously reported from Uganda (12). Previous data on seasonality showed influenza activity peaking from May to November with most cases appearing in October and November (12). However, in our study, influenza cases declined to less than 10% from May through December 2022 (Fig. 2). Rather, the highest number of influenza cases occurred in March and April 2022. Such variable and limited information regarding influenza in certain regions of the world emphasizes the need for a surveillance system to track influenza infections and their spread. As shown here, the timing of respiratory infections can change during a pandemic because of measures to reduce the spread of similarly transmitted pathogens (8). These patterns demonstrate the utility of detecting and continuously tracking respiratory pathogens throughout the country.

Another objective of our surveillance program was to understand SARS-CoV-2 variants in the community by continuously sampling ILI/SARI patients reported from a defined catchment area. To fulfill this objective, SARS-CoV-2-positive samples from ILI/SARI sites and other health centers outside the surveillance program, and with CT values < 30, were sequenced on the Illumina MiSeq platform to determine the circulating variants (Fig. 4). We successfully sequenced and got consensus genomes from 146 SARS-CoV-2 samples, which were deposited in GISAID (accession numbers are found in Table 1). All the variants detected from January to December 2022 at both surveillance sites and other community health centers were Omicron. Omicron was first identified in November 2021 and quickly spread, reaching the United States in December 2021 (20). The sentinel surveillance sites used here were able to pick up Omicron variants. There were 10 samples from surveillance sites and 12 samples from other community health centers that were collected from patients in January 2022. The vast majority of these were BA.1.1 (20/22).

Our study also reports co-infections in both out-patients and hospitalized patients with a predominance of out-patient samples. The sensitivity and robustness of our surveillance program demonstrate its ability to detect influenza cases when COVID-19 activity was at its peak in January and June 2022. The trends in the rise and fall of COVID-19 cases in Uganda, seen in our network, coincided with the national trends. Other studies have reported co-infections of SARS-CoV-2 and influenza globally (21 – 25). Co-infections of SARS-CoV-2 and influenza have also been highlighted as a cause of concern due to the demonstrated worsening of the clinical picture in such cases. A study in Saudi Arabia reported mortality and ICU admissions in influenza A/SARS-CoV-2 co-infections (24), and triple co-infections with influenza A and B, and SARS-CoV-2 have also been reported (26).

To the best of our knowledge, this is the first report from Uganda on SARS-CoV-2/influenza co-infection cases identified through sentinel surveillance in a community setting. Our efforts of parallel testing for influenza and SARS-CoV-2 are in line with WHO recommendations for integrated testing for influenza and COVID-19. We report the circulation of influenza viruses in the community as well as hospital settings across the entire country, even during the COVID-19 pandemic, with AH3 being the dominant influenza virus A subtype that circulated in Uganda in 2022. Overall, this study reports a low prevalence of co-infections of SARS-CoV-2 and influenza.

There are advantages to using specified surveillance sites for monitoring infectious diseases throughout Uganda. When other health centers in the broader community that are not in the surveillance network send samples for processing, there are many instances of not receiving appropriate metadata that is important for decision-making, such as patient demographics or location-specific data. At surveillance sites, this can be better controlled, and the right metadata can be collected for public health officials to track disease spread and outbreak progression in the country. The lack of metadata also makes it hard to share with the scientific community, for example, uploading to GISAID. Another benefit of surveillance sites is sample integrity. We ensure the samples are kept frozen, and the cold chain is maintained until they get to the laboratory and stored at −80°C until processing. The surveillance sites have more control over the whole process from sample collection to sequencing. Other community samples were often delivered at room temperature in envelopes and therefore some samples failed to give good results.

This study had limitations. We present data from only 1 year up to Epi week 50 when SARS-CoV-2 genomic sequencing was integrated into ILI/SARI surveillance. We initiated testing only influenza viruses with SARS-CoV-2 and have not included other respiratory viruses, particularly RSV. However, our study reports SARS-CoV-2 genomic data from a geographically distributed network of 16 influenza sentinel sites throughout Uganda as well as other health centers outside the ILI/SARI sentinel surveillance network. In the future, additional respiratory viruses can be tested for at these already established surveillance sites.

As the world progresses toward COVID-19 endemicity, it will be important to strengthen surveillance capacities for other respiratory pathogens, which may lead to significant morbidity and mortality, and overburden healthcare systems in the future. Therefore, adopting and implementing cost-effective and reliable testing methods for the diagnosis of respiratory viruses with epidemic and pandemic potential will help in monitoring the trends and seasonality of those viruses for the prevention of epidemics and pandemics. Uganda will continue to benefit from the established sentinel surveillance site network throughout the country, not only for influenza surveillance but also for other respiratory pathogens and future pandemic pathogens.

## Data Availability

The SAR-CoV-2 genomes describe here are available on GISAID (https://www.gisaid.org) under accession numbers listed in Table 1.
